# MicroRNA26 attenuates vascular smooth muscle maturation via endothelial BMP signalling

**DOI:** 10.1371/journal.pgen.1008163

**Published:** 2019-05-15

**Authors:** Charlene Watterston, Lei Zeng, Abidemi Onabadejo, Sarah J. Childs

**Affiliations:** Alberta Children's Hospital Research Institute and Department of Biochemistry and Molecular Biology, Cumming School of Medicine, University of Calgary, Calgary AB, Canada; University of Pittsburgh, UNITED STATES

## Abstract

As small regulatory transcripts, microRNAs (miRs) act as genetic ‘fine tuners’ of posttranscriptional events, and as genetic switches to promote phenotypic switching. The miR *miR26a* targets the BMP signalling effector, *smad1*. We show that loss of *miR26a* leads to hemorrhage (a loss of vascular stability) *in vivo*, suggesting altered vascular differentiation. Reduction in *miR26a* levels increases *smad1* mRNA and phospho-Smad1 (pSmad1) levels. We show that increasing BMP signalling by overexpression of *smad1* also leads to hemorrhage. Normalization of Smad1 levels through double knockdown of *miR26a* and *smad1* rescues hemorrhage, suggesting a direct relationship between *miR26a*, *smad1* and vascular stability. Using an *in vivo* BMP genetic reporter and pSmad1 staining, we show that the effect of *miR26a* on smooth muscle differentiation is non-autonomous; BMP signalling is active in embryonic endothelial cells, but not in smooth muscle cells. Nonetheless, increased BMP signalling due to loss of *miR26a* results in an increase in *acta2*-expressing smooth muscle cell numbers and promotes a differentiated smooth muscle morphology. Similarly, forced expression of *smad1* in endothelial cells leads to an increase in smooth muscle cell number and coverage. Furthermore, smooth muscle phenotypes caused by inhibition of the BMP pathway are rescued by loss of *miR26a*. Taken together, our data suggest that *miR26a* modulates BMP signalling in endothelial cells and indirectly promotes a differentiated smooth muscle phenotype. Our data highlights how crosstalk from BMP-responsive endothelium to smooth muscle is important for smooth muscle differentiation.

## Author summary

The structural integrity of a blood vessel is critical to ensure proper vessel support and vascular tone. Vascular smooth cells (vSMCs) are a key component of the vessel wall and, in their mature state, express contractile proteins that help to constrict and relax the vessel in response to blood flow changes. vSMCs differentiate from immature vascular mural cells that lack contractile function. Here, we use a zebrafish model to identify a small microRNA that regulates vascular stabilization. We show that a small regulatory RNA, microRNA*26a* is enriched in the endothelial lining of the blood vessel wall and, through signalling, communicates to the smooth muscle cell to control its maturation. Providing a mechanistic insight into vSMC differentiation may help develop and produce feasible miR-based pharmaceutical to promote SMC differentiation.

## Introduction

Vascular smooth muscle cells (vSMCs) provide structural integrity to the vessel wall. Guided control of signalling cascades, including Platelet derived growth factor (Pdgf), Notch, and Transforming Growth Factor-β/Bone morphogenic Protein (TGF-β/BMP) recruits and induces differentiation of perivascular mural cells (vSMCs and pericytes) to create a two-layered vessel wall with an internal endothelial cell lining and a muscle cell covering [1–3]. Once the vSMCs surround the vessel, they begin depositing extracellular matrix (ECM) proteins Laminin, Collagen IV and Fibulins to support the vessel wall [4]. vSMCs then take on a mature phenotype that stabilizes the underlying endothelial cells through induction of quiescence, expression of junctional and attachment proteins, and expression of contractile proteins to provide myogenic tone [2,5–7].

vSMCs maintain phenotypic plasticity and can undergo a phenotypic switch from a quiescent contractile state to a proliferative synthetic state in response to cellular stimuli [4,8]. Contractile vSMCs are defined by an elongated and thin ‘spindle-shaped’ morphology and low rates of proliferation. The expression of key differentiation markers such as smooth muscle (α)-actin (Acta2), smooth muscle β-myosin heavy chain (Myh11), and transgelin (Sm22α) allows vSMCs to perform their contractile function and provide vascular tone. In contrast, the immature synthetic vSMCs have reduced expression of contractile genes, produce ECM proteins, are highly proliferative, and have a rhomboid or rounded morphology [9–12].

Numerous studies have demonstrated that BMP signaling through Smad1 modulate vSMC plasticity (reviewed by [13]). Defective BMP signalling can affect both endothelial and vSMC cells [14–21]. Aberrant vSMCs phenotype switching plays a critical role in the pathogenesis of vascular diseases such as hereditary hemorrhagic telangiectasia (HHT) and pulmonary arterial hypertension (PAH). In canonical Smad-mediated BMP signaling, Smad1 is phosphorylated by the serine-threonine kinase activity of a type 1 BMP receptor (ACVRL1 (ALK1)/ BMPR1A, BMPR1B) allowing it to associate and dimerize with the co-mediator Smad4 and translocate to the nucleus to control gene transcription. Murine homozygous null mutants for BMPR-1a (Activin like kinase 3, ALK3) or the type II receptor BMPR-2 (which is mutated in human patients with PAH) [22,23] and their ligand Bmp4 or downstream co-Smad4 are embryonic lethal, and present with vascular deformities attributable to a loss of Smad1 mediated signalling [24]. Mutations in ALK1 lead to HHT2, a disease characterized by arteriovenous malformations (AVMs) [25]. Deletion of Alk1 in mice leads to cranial hemorrhages, AVM-like fusion of micro-vessel plexi, dilation of large vessels and reduced coverage of vessels by vSMCs [26]. In zebrafish, disruption of Alk1 signalling results in pathological arterial enlargement and maladaptive responses to blood flow that generate AVMs. Potential vSMCs defects in this model have not been assessed [27].

As small noncoding RNAs, microRNAs (miRs) regulate gene expression of key vSMC marker genes to control vSMC dynamics. [28–31]. A number of miRs have been identified as modulators of the vSMC phenotype *in vitro* and *in vivo*, including *miR-145*, *miR-21*, *miR-221*, *miR-222* and *miR-146a* [32–40]. We previously showed that miR-145 promotes visceral smooth muscle differentiation via controlling cross-talk between epithelial cells and smooth muscle [32,41].

Here, we investigate the role of *microRNA26a* (*miR26a*) in regulating vSMC dynamics using the zebrafish model of vessel stabilization. *miR26a* regulates proliferation, migration and differentiation of vSMCs and has been shown to target *smad1*, a key intracellular mediator of BMP signalling, in cultured vSMCs *in vitro* [42–45].

*miR26a* expression is altered during abdominal aortic aneurysm (AAA) and neointimal lesion formation [43,45]. However, the role of *miR26a in vivo* in an intact animal in the context of developing vSMCs are largely unknown. Using a combination of genetic gain and loss of function methods to understand the role of *miR26a in vivo*, we show that *miR26a* acts within a BMP responsive pathway to fine tune vSMC maturation via targeting *smad1*. Interestingly, we find that active BMP signalling and changes in Smad1 activation are observed within endothelium *in vivo*, and not in smooth muscle cells. Together the evidence suggests that *miR26a* plays a role in regulating blood vessel stabilization via a non-autonomous mechanism.

## Results

### *miR26a* is expressed in developing blood vessels

**Fig 1:**
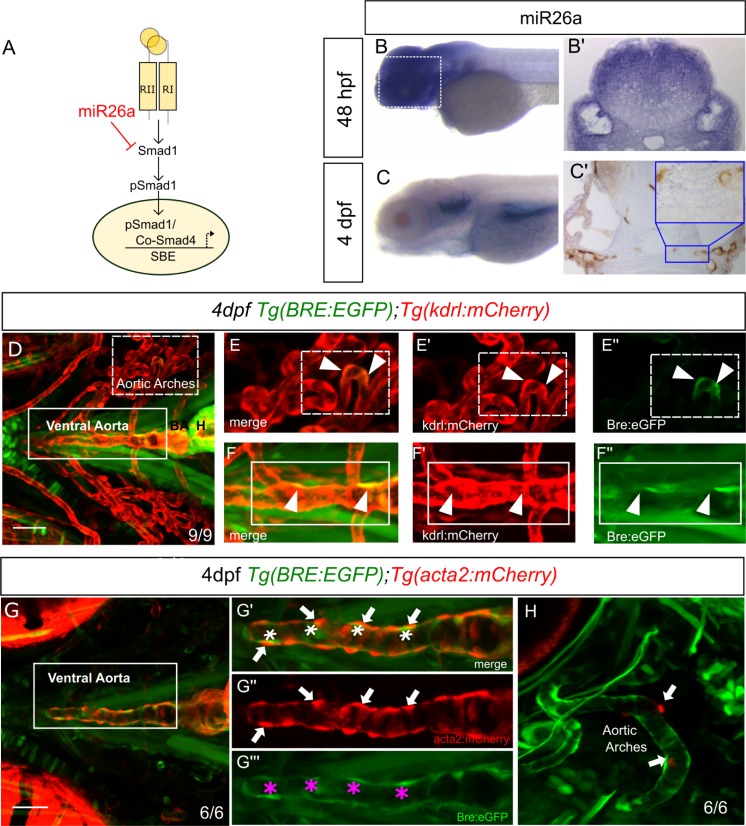
*miR26a* is expressed in blood vessels; endothelial cells have active BMP signalling. A) Model of how *miR26a* controls BMP signaling via direct targeting of *smad1*. B) Lateral view of whole mount in situ expression of *miR26a* at 48 hpf shows ubiquitous expression pattern, with strong expression in the ventral head of the embryo. B’) Cross section of the head at 48 hpf. C) At 4 dpf *miR26a* is expressed in the pharyngeal arches, bulbous arteriosus and ventral aorta. C’) Cross section of the head showing *miR26a* expression in blood vessels (purple; punctate stain) compared with endothelial stain (brown; kdrl:GFP transgenic). Inset is an enlargement of image in C’. D) Ventral view of the pharyngeal region of a 4 dpf double transgenic *Tg(BRE*:*EGFP);Tg(kdrl*:*mCherry)* embryo shows BRE:EGFP (green) expression within endothelial cells in aortic arches (red, white arrowheads in E’-E”‘) and ventral aorta (red, white arrowheads F’-F”‘). G-H) Ventral and lateral views of a 4 dpf double transgenic *Tg(BRE*:*EGFP); Tg(acta2*:*mCherry)* zebrafish shows that *acta2* positive cells are in direct contact with BMP-responsive endothelial cells but do not express BRE:EGFP. Scale bar represents 50μm.

*miR26a* targets *smad1* and thereby directly regulates BMP signalling ([36,43] Fig 1A). To observe the spatial gene expression pattern of *miR26a* in developing embryos we used *in situ* hybridization. At 48 hpf, *miR26a* has a ubiquitous expression pattern (Fig 1B and 1B’), however by 4 dpf expression becomes enriched in the ventral head of the embryo, with strong expression in the pharyngeal region, bulbus arteriosus and ventral aorta (Fig 1C). *miR26a* is expressed in and around the blood vessel endothelium where it could potentially play a role modulating BMP signalling in blood vessels (compare to *kdrl*: *GFP* stain; Fig 1C’, inset). In order to further analyze the cell specific expression of *miR26a*, we used fluorescent-activated cell sorting (FACS) to isolate EGFP^+ve^ vSMCs and mCherry^+ve^ endothelial cells from 4 dpf *Tg(acta2*:*EGFP;kdrl*:*mCherry)* embryos. In keeping with the *in-situ* hybridization data, RT-qPCR showed that *miR26a* is indeed expressed in both cell types, although it is not significantly enriched in endothelial cells (S1 Fig). FACS sorting efficiently separated vSMCs and endothelial cells; we find that *acta2*: *EGFP*^+ve^ vSMCs cells have an average 37.4-fold enrichment in *acta2* expression, and minimal expression of *alk1* or *smad1* when compared to *kdrl*: *mCherry* endothelial cells. However, *smad1* is 14-fold enriched and *acvrl1* is 3.5-fold enriched in *mCherry*^+ve^ endothelial cells while there is nearly no expression of *acta2* (S1 Fig). Thus, a *miR26a* target, *smad1* is enriched in endothelial cells.

### BMP signalling is active in the aorta endothelium

We next tested the relationship between *mir26a* expression and activated BMP signaling using an *in vivo* reporter of Smad1/5 activity. *Tg(BRE*:*EGFP]* transgenic fish encode EGFP driven by an upstream *Bmp Response Element (BRE)* that contains multiple short Smad-binding sites from the *id1* promoter, a major transcriptional target of canonical Bmp/Smad1 signaling [46,47]. We crossed *Tg(BRE*:*EGFP]* to endothelial *Tg*(*kdrl*:mCherry*)* or vSMC *Tg(acta2*:*mCherry)* lines to observe BMP activation in endothelial and vSMCs, respectively (Fig 1D and 1G). We use the 4 dpf time point as vSMC cells first differentiate and begin to express the mature marker *acta2* between 3 and 4 dpf [7,48]. Surprisingly, although *miR26a* has been implicated in controlling Smad1 regulated vSMC dynamics directly, we found that transgenic *BRE*:*EGFP* signals are restricted to the endothelium of the vessel wall and have co-localized expression with *kdrl*:*mCherry* (Fig 1E, 1E’, 1F and 1F’). The *acta2*:*mCherry*-positive vSMCs lie directly adjacent to *BRE*:*EGFP*-expressing cells, with no detectable expression of *BRE*:*EGFP* in vSMCs on the ventral aorta or in pharyngeal aortic arch arteries (Fig 1G, 1G” and 1H, both ventral and lateral projections are shown). Similarly, *acta2*:*mCherry*-positive cells are closely associated with pSmad1-positive endothelial cells but do not show pSmad1 staining (S1D Fig). Together, our data suggest that in early development, *miR26a* and *smad1* are expressed within endothelial cells where BMP signaling is also active, as visualized by two methods of detection.

### Loss of *miR26a* leads to upregulation of *smad1* mRNA

**Fig 2:**
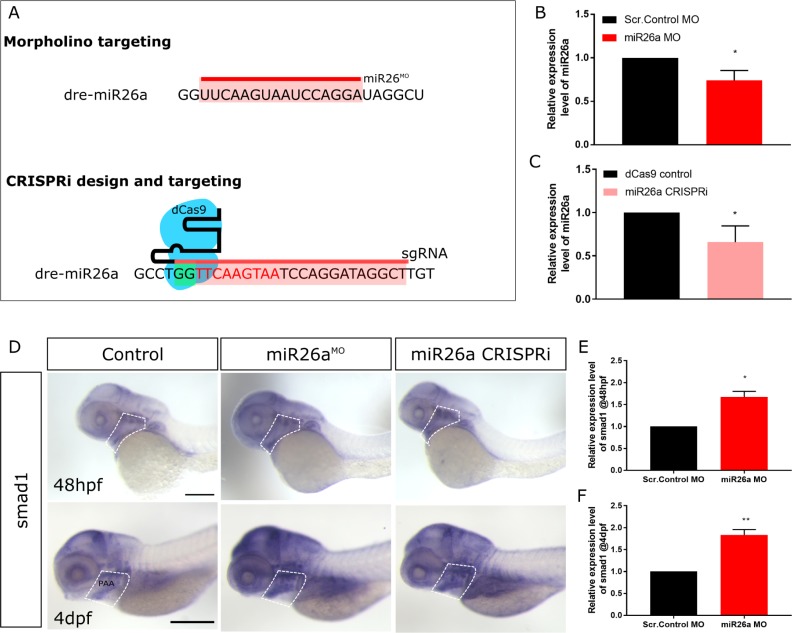
*miR26a* knockdown increases *smad1* expression. A) Schematic of *miR26a* transient knockdown methods. B and C) Relative expression level of *miR26a* in morpholino and CRISPRi injected embryos at 48 hpf (n = 3). D) Whole mount in situ hybridization staining for *smad1* at 48 hpf and 4 dpf shows increased expression of *smad1* in *miR26a* knockdown embryos particularly in the ventral aorta, aortic arches and pharyngeal region (dotted outline). Scale bar represents 200μm. E) Relative expression of *smad1* in 48 hpf morphants is increased compared to control embryos (n = 3). F) Relative expression of *smad1* in 4 dpf *miR26a* morphants is increased compared to control embryos (n = 4). RT-qPCR data show the mean ± SEM, Student's two-tailed t-test *p < 0.05, n = number of biological replicates.

The highly conserved miR-26 family constitutes *miR26a-1*, *miR26a-2*, *miR26a-3* and *miR26b* [44] as identified by their seed sequences and accessory sequence. In zebrafish and humans, *miR26a-1*, *miR26a-2* and *miR26a-3* have the same mature sequence, and only differ from the mature *miR-26b* sequence by two nucleotides [42,49]. To investigate the role of *miR26a* in vascular development *in vivo*, we knocked down *miR26a* using an antisense morpholino that targets the mature miRNA seed sequence of all three *miR26a* isoforms. A 6bp mismatch scrambled control morpholino was used as a control. 1 ng doses of morpholino were used, as suggested by current guidelines [50,51]. In parallel, we designed a second genetic knockdown approach using CRISPR interference (CRISPRi) [52] to target the pri-miR hairpin structure using the complementary sequence to the mature miRNA (Fig 2A). RT-qPCR shows a 26% (0.74±0.65) reduction in *miR26a* following *miR26a* MO knockdown and 34% (0.65±0.10) reduction of *miR26a* using CRISPRi (Fig 2B and 2C) confirming that both knockdown methods result in decreased *miR26a* expression. *smad1* is a demonstrated target of *miR26a in vitro* [36,43]. In support of *smad1* being a *miR26a* target *in vivo*, *miR26a* knockdown results in increased *smad1* expression in 48 hpf and 4 dpf injected embryos as compared to controls by in situ hybridization (Fig 2D) and resulted in an average 1.6-fold and 1.8-fold increase by RT-qPCR, respectively (Fig 2E and 2F).To further determine whether *miR26a* can regulate *smad1* expression *in vivo*, we designed a sensor assay and fused the *smad1* 3′UTR to EGFP (*EGFP*: *smad1pA*). This was co-injected with an internal mCherry control into single-cell zebrafish embryos in the presence or absence of a *miR26a* morpholino. When fluorescence levels were examined at 24 hpf, injections of *EGFP*: *smad1pA* sensor mRNA alone resulted in EGFP expression; however, this fluorescence was enhanced by over 65% by co-injection of *miR26a* morpholino (S2A and S2B Fig).

At 4 dpf, upregulation of *smad1* in *miR26a* morphants and CRISPRi knockdown embryos is more prominent in the ventral pharyngeal region, with staining in the ventral aorta, aortic arches and bulbous arteriosus (Fig 2D, highlighted areas), similar to where *miR26a* is expressed most strongly (Fig 1B and 1C). In a complementary approach, we injected a *miR26a* mimic to overexpress *miR26a* and observed an increase in *miR26a* expression by RT-qPCR (S2C Fig), as well as a marked reduction of *smad1* expression in the ventral pharyngeal region by in situ hybridization (S2E Fig). Overexpression of *miR26a* results in mildly dorsalized embryos by 48 hpf with pericardial edema, dorsal axis defects and poor circulation (S2D Fig), suggesting overexpression of *miR26a* disrupts the BMP pathway that patterns early embryonic axes.

### Loss of *miR26a* leads to increased phosphorylated Smad1 in endothelium

**Fig 3:**
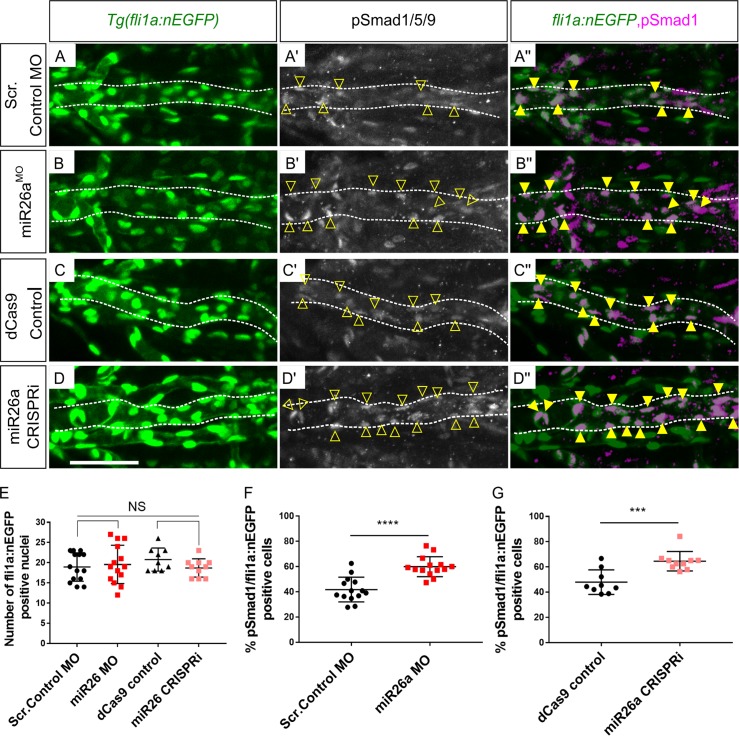
*miR26a* knockdown embryos have increased endothelial pSmad1. A-D) Ventral view confocal projections of the 4 dpf ventral aorta (dotted outline). Endothelial nuclei (*fli1a*:*nEGFP*; A-D, arrowheads) and pSmad1/5/9 (pSmad1,white A’- D’) and overlay (magenta, A”- D”) in 4 dpf Scr. Control (A), *miR26a* MO (B), dCas9 control (C) and *miR26a* CRISPRi (D) embryos. Yellow arrowheads indicate double positive pSmad1 + *fli1a*:*nEGFP* nuclei in the ventral aorta. E) Quantification of total number of *fli1a*:*nEGFP* nuclei in the ventral aorta. F-G) Quantification of the percentage of double pSmad1; *fli1a*:*nEGFP* positive nuclei in *miR26a* morphants (F) and miR26a CRISPRi (G) embryos. N = 3 experiments. Total embryos are as follows: Scr. Control MO n = 14, *miR26a* MO n = 14, dCas9 control n = 9 and *miR26a* CRISPRi n = 10. Student's two-tailed t-test, p***< 0.0001 and p ****< 0.00001 as compared to WT, error bars = SD. Scale bar: represents 50μm.

We next tested whether the increased expression of *smad1* mRNA in *miR26a* knockdown embryos leads to enhanced Smad1 phosphorylation. Wildtype immunostaining showed pSmad1/5/9 is high in endothelium but not in vSMCs (S1D–S1D” Fig). *miR26a* knockdown embryos do not show any significant difference in endothelial cell number as compared to controls, using endothelial nuclear transgenic lines (*Tg(fli1a*:*nEGFP; kdrl*:*mCherry);* Fig 3A–3E, and S3 Fig). However, there is a significant 20% increase in pSmad1 positive/ *fli1a*:*nEGFP* nuclei in *miR26a* knockdown embryos as compared to controls, with an average of 60±3.1% and 64.9±3.9% in *miR26a* morphants (Fig 3B–3B” and 3F) and CRISPRi embryos (Fig 3D, 3D” and 3G), respectively as compared to 41±2.9 and 46±6.5% in controls.

### Increased levels of *smad1* lead to vascular stability defects

**Fig 4:**
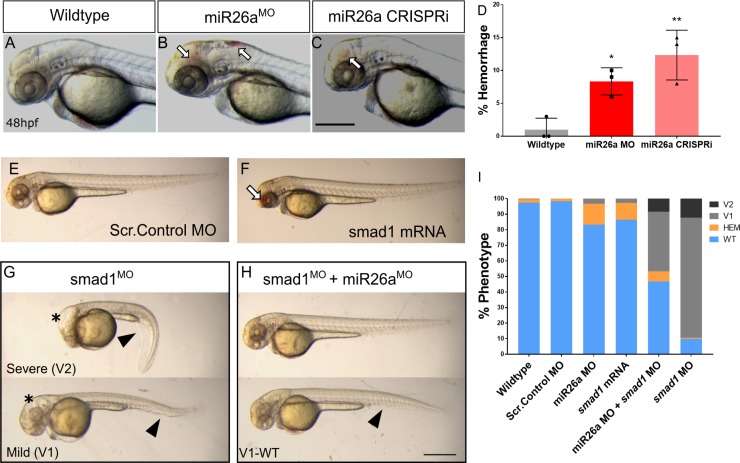
Increased levels of *smad1* result in defects in the vascular system and body axis. A-C) Representative 48 hpf *miR26a* knockdown embryos with hemorrhage, as indicated by arrows. D) Quantification of average rates of hemorrhage. (Error bars = SD Unpaired t test, *miR26a* MO *p< 0.01 and mi26 CRISPRi **p< 0.001 as compared to WT, N = 3, Wildtype n = 224, miR26a MO n = 124, miR26a CRISPRi n = 180). E-F) Representative morphology after *smad1* overexpression. G-I) *miR26a* and *smad1* double knockdown experiments. G) Representative 48 hpf *smad1* MO embryos with mild (V1) and severe (V2) ventralization phenotypes. H) Representative 48 hpf double *miR26a* and *smad1* knockdown embryos with rescued hemorrhage and normal body axis showing only mild (V1-WT) ventralization phenotypes. I) Quantification of observed phenotypes double knockdown experiments (N = 4, total n wildtype = 193, Scr. Control MO = 157, *smad1* MO = 175, *smad1* mRNA = 95, *miR26a* MO = 190, and *miR26a* MO + *smad1* MO = 190. One Way ANOVA of hemorrhage phenotype; Wildtype/Scr. Control MO vs. *miR26a* MO p< 0.0001 Wildtype/Scr. Control vs. *SMAD1* mRNA p< 0.0001 *miR26a* MO vs. *miR26a* MO+ *smad1* MO p< 0.0001 One Way ANOVA of V1 phenotype: Wildtype/Scr. Control MO; vs. *smad1* MO p< 0.0001 vs. *miR26a* MO+ *smad1* MO p< 0.0001. Error Bars = SEM. Scale bar represents 500μm.

Loss of *miR26a* leads to compromised vessel integrity at 48 hpf. *miR26a* morphants have an average 13±2% hemorrhage (Fig 4B and 4D) and CRISPRi embryos have an average 15±1% hemorrhage (Fig 4C and 4D) as compared to 2–3% rate of the controls. The phenotype is dose-dependent as higher doses of morpholino lead to an increase in hemorrhage to 40% and a 1.8-fold reduction in *miR26a* expression (S4C and S4D Fig). As *smad1* overexpression has not been previously connected to vascular stability defects, we next tested whether overexpressed *smad1* could lead to hemorrhage. Injection of *smad1* mRNA into single cell stage embryos resulted in significantly higher hemorrhage rate of 12±0.9% in injected embryos as compared to uninjected controls (Fig 4E, 4F and 4I).

Further, as *miR26a* knockdown leads to increased *smad1* levels, we predicted that reduction in *smad1* would rescue hemorrhage in *miR26a* knockdown embryos. Double knockdown by co-injection of *smad1* [53] and *miR26a* morpholinos reduced hemorrhage rates to below 5±0.8% (Fig 4H, top embryo and Fig 4I). Of note, *smad1* MO alone did not result in hemorrhage; however it did result in a range of phenotypes associated with *smad1* knockdown including dorsal-ventral axis defects and hydrocephalus as previously reported [53]. *smad1* knockdown led to an average a 77±8.6% of embryos with a mild (V1) ventralized defect and 12±17% with a more severe (V2) phenotype (Fig 4G–4I). Both defects were reduced in double knockdown embryos (Fig 4G and 4H, bottom). Thus, reducing *miR26a* or increasing *smad1 in vivo* leads to leads to a loss of vascular stability and hemorrhage.

### Loss of *miR26a* leads to increased numbers of *acta2*-positive vSMCs and upregulation of pathways involved in endothelial-vSMC crosstalk

**Fig 5:**
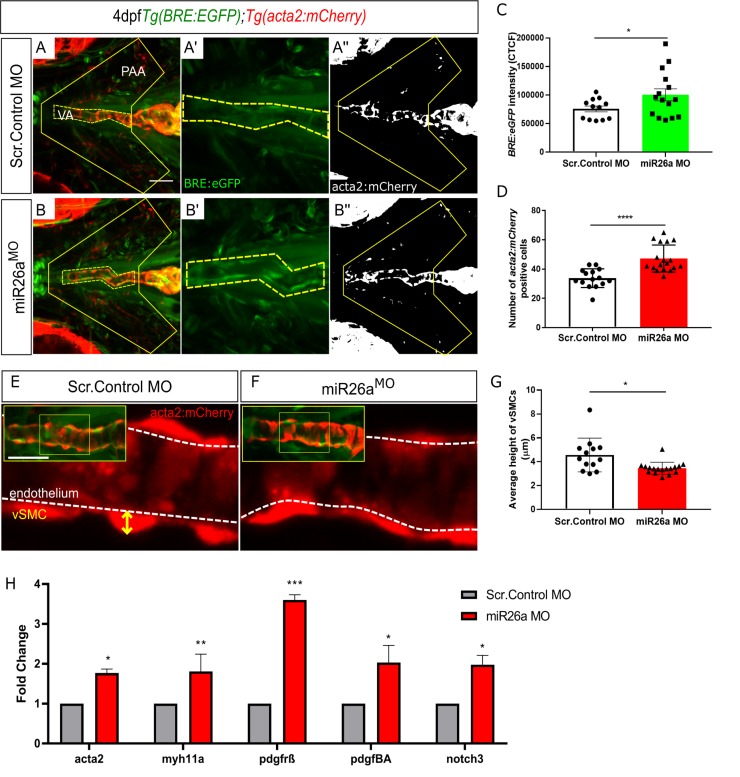
Loss of *miR26a* morphants leads to increased expression of vSMC genes and *acta2*-positive vSMCs. A-B) Representative ventral views of 4 dpf *Tg(BRE*:*EGFP); Tg(acta2*:*mCherry)* embryos. Scr. Control embryos (A-A”) and *miR26a* morphant embryos (B-B”) showing qualitative upregulation of *BRE*:*EGFP* in the ventral aorta (VA) and pharyngeal arch arteries (PAA). C) Quantification of green fluorescent marker (*BRE*:*EGFP*) along the VA, taken from the highlighted yellow region in A’ and B’, and represented as corrected total cell fluorescence (CTCF) (N = 3, *miR26a* MO n = 15, Scr. Control n = 12, Unpaired t test, ****p< 0.0001 as compared to control, error bars = SEM). D) Quantification of *acta2* positive cell number on VA and PAAs, within area outlined in A” and B”. Number of *acta2* positive cells is significantly increased in *miR26a* morphants (N = 3, miR26a MO n = 18, Scr. Control n = 15, Unpaired t test, ****p< 0.0001 as compared to control, error bars = SEM). E and F) Measurement of vSMC height (yellow axis) from the endothelium (white dashed line). Representative images of ventral aorta (from insets), Scr. Control (E) and *miR26a* morphants (F). G) Quantification of average vessel heights along the length of the VA (N = 3, miR26a MO n = 18, Scr. Control n = 13, Student's two-tailed t-test, ****p< 0.0001 as compared to control, error bars = SEM). H) RT-qPCR quantification of vSMC differentiation genes in injected controls and *miR26a* morphants (n = 3). RT-qPCR data show the mean ± SEM, Student's two-tailed t-test *p < 0.05, n, number of biological replicates.

To demonstrate functional consequences of increased endothelial BMP signalling on vSMCs, we next investigated vSMC investment on the ventral aorta and pharyngeal arch arteries of *Tg(BRE*:*EGFP;acta2*:*mCherry)* embryos in *miR26a* knockdown embryos. This assay allowed us to make three key observations. Firstly, *BRE*:*EGFP* signal intensity is enhanced in *miR26a* morphants (Fig 5A’, 5B’ and 5C), which correlates with the increased pSmad1 staining we observed in endothelial nuclei of knockdown embryos (Fig 3). Secondly, the number of *acta2*:*mCherry* positive cells along the ventral aorta and pharyngeal arch arteries (PAA) is increased in *miR26a* knockdown embryos (33.8±1.6 in controls vs 47.2 ±2.2 in *miR26a* knockdown, Fig 5A”, 5B” and 5D). Thirdly, the increase in *acta2* positive cell number is accompanied by a change in cell morphology in *miR26a* knockdown embryos (Fig 5E–5G). In control embryos, *acta2* positive vSMCs have a rounded, punctate morphology, and ‘sit’ high on the vessel wall with an average height of 4.5±0.4 μm, above the underlying endothelium. In *miR26a* morphants, vSMCs are have a significantly reduced vSMC height of 3.4 ± 0.1μm, and appear flatter and more closely apposed to the endothelium when compared to control embryo vSMCs. These data suggest that loss of *miR26a* results in increased vSMC coverage along blood vessels and a shift to a differentiated morphology.

In parallel, we quantitated gene expression for vSMC differentiation genes. RT-qPCR using isolated embryonic head mRNA at 4 dpf showed a 1.7-fold increase in *acta2* and 1.8-fold increase in *myh11a* mRNA in *miR26a* morphants (Fig 5H). Further, using in situ hybridization, we found that *miR26a* morphants have increased expression of *acta2* and *myh11a* in the pharyngeal region (S5 Fig), similar to the location of increased *smad1* staining (Fig 2D). Conversely by 4 dpf, *miR26a* mimic injected embryos had reduction in *acta2* and *sm22* expression by in situ hybridization (S5 Fig). The Bmp/ Notch3/ Pdgf signalling axis is an important regulator of vSMC proliferation and subsequent differentiation. We found that the vSMC notch receptor *notch3* has a 2.0-fold increase in *miR26a* morphants (Fig 5H). Furthermore, the endothelial expressed ligand *pdgfba* and its mural cell receptor *pdgfr*β, had a 3.6 and 2.0-fold increase, respectively, in *miR26a* morphants as compared to controls. This suggests that increased vSMC numbers could potentially arise from enhanced proliferation via activation of the Pdgfrβ pathway downstream of Smad1 activation. Increases in *acta2*, *myh11* and *notch3* may therefore reflect increased cell numbers in addition to increased vSMC differentiation.

### Endothelial overexpression of smad1 promotes vSMC differentiation

**Fig 6:**
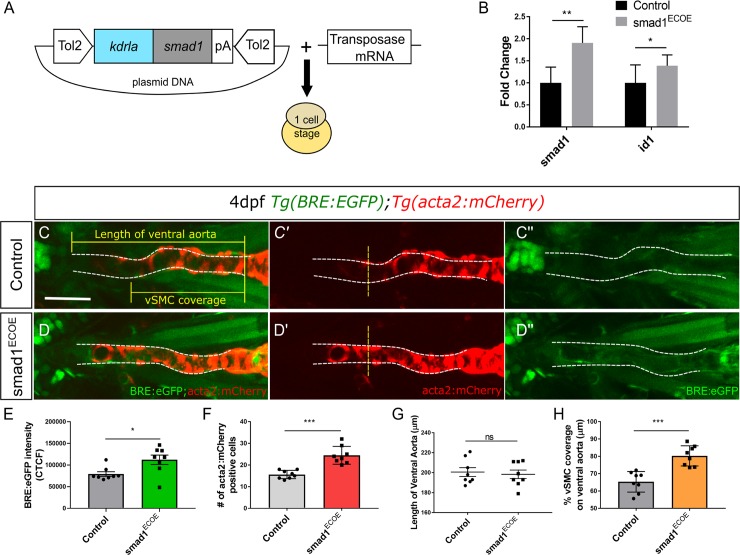
*smad1* overexpression in endothelial cells results in increased vSMC coverage. A) Vector construct for overexpression of *smad1* under the endothelial cell promoter *kdrla*. B) RT-qPCR fold change in *smad1* and *id-1* expression levels in endothelial specific *smad1* overexpressing embryos (smad1^ECOE^) embryos at 4 dpf (n = 3). RT-qPCR data show the mean ± SEM, Student's two-tailed t-test *p < 0.05, n, number of biological replicates. C–D) Representative orthogonal projections of ventral views of 4 dpf *Tg(BRE*:*EGFP)*; *Tg(acta2*:*mCherry)* embryos. Control embryos (C-C”) and *smad1*^ECOE^ embryos (D-D”) showing endothelial BRE:EGFP and vSMC acta2:mCherry expression in the ventral aorta (VA) and pharyngeal arch arteries (PAA). E) Quantification of green fluorescent marker (*BRE*:*EGFP*) along the VA, highlighted within the yellow region in C” and D”, as corrected total cell fluorescence (CTCF). F) Quantification of *acta2* positive cell number on VA, within area outlined in C and D. Number of *acta2* positive cells is significantly increased in *smad1*^ECOE^ embryos. G) Quantification of length of VA, within area outlined in C and D. H) Quantification of the percent vSMC coverage of ventral aorta. For each quantification, N = 3, *smad1*^ECOE^ embryos n = 8, Control n = 8, Student's two-tailed t-test, *-***p< 0.01–0.0001 as compared to control. Error bars = SEM, Scale bar represents 50μm.

To demonstrate that *smad1* expression in endothelial cells promotes vSMC differentiation, we expressed *smad1* under an endothelial promoter in a transposon vector (TolCG2:*kdrl*:*smad1*, hereafter *smad1*^*ECOE*^; Fig 6A). The vector and transposase or transposase alone control were injected into *Tg(BRE*:*EGFP;acta2*:*mCherry)* embryos and scored at 48 hpf and 4 dpf. At 48 hpf, 10% of smad1^ECOE^ embryos hemorrhage, similar to the increased hemorrhage observed in mirR26 knockdown and global *smad1* mRNA overexpression (Fig 4). Higher doses of the vector result in significant cranial and pericardial edemas (S6A and S6B Fig). At 4 dpf, RT-qPCR of *smad1*^*ECOE*^ embryos shows a 1.9-fold increase in *smad1* and 1.4-fold increase in the BMP responsive gene *id1* expression as compared to control (Fig 6B). Similarly, *BRE*:*EGFP* fluorescence is also increased in *smad1*^*ECOE*^ embryos by 30% as compared to controls (Fig 6C”, 6D” and 6F). Together, the data show that activation of Smad1 was significantly increased in *smad1* injected embryos. *smad1*^*ECOE*^ embryos do not show a change in the length of the ventral aorta, however the *BRE*:*EGFP* signal extends further along the ventral aorta (Fig 6C”, 6D” and 6G). The total number of *acta2*:*mCherry* positive vSMCs along the ventral aorta in *smad1*^*ECOE*^ embryos was significantly increased from 15±0.6 in controls to an average 24±1.4 cells (Fig 6C’, 6D’ and 6E). The percent vSMC coverage of the ventral aorta is also increased by 20% with an average of 80 ± 2.05% in *smad1*^*ECOE*^ as opposed to 65.3±2.1% in controls (Fig 6C’, 6D’ and 6H). Our data suggests that upregulation of *smad1* in endothelial cells is sufficient to increase vSMC number and coverage of the ventral aorta of 4 dpf embryos.

### BMP inhibition rescues the effect of on vSMC differentiation after *miR26a* knockdown

**Fig 7:**
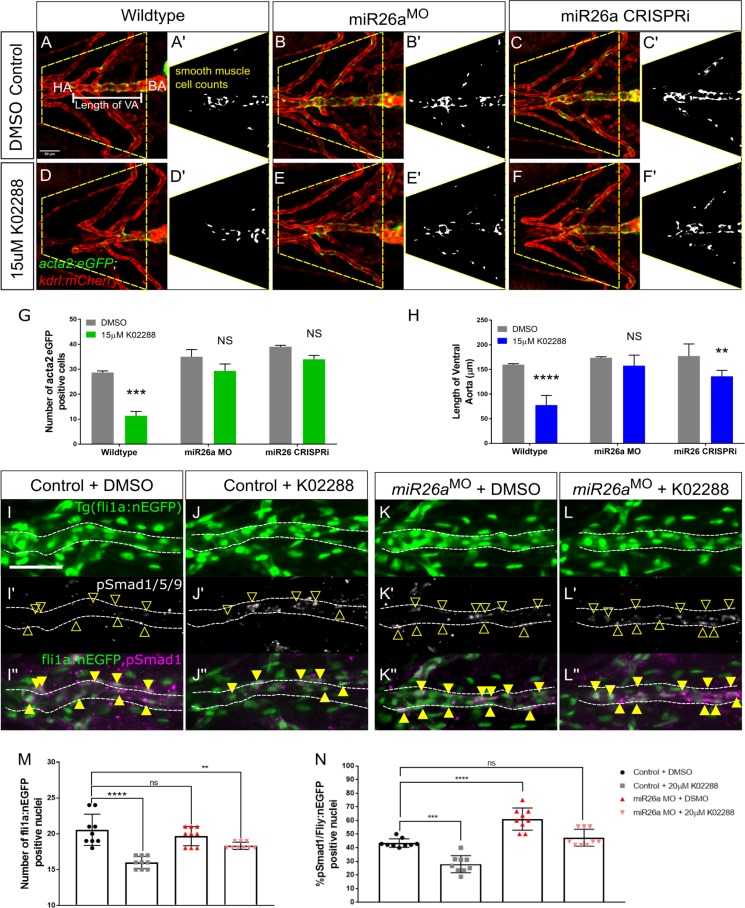
*miR26a* controls vSMC differentiation via *smad1*-mediated BMP signaling. Ventral aorta showing endothelial (red) and smooth muscle (green) cells in *miR26a* morphants or CRISPRi- injected embryos treated with vehicle control (DMSO) or 15μM K02288 from 52 hpf to 4 dpf. A-C) DMSO-treated vehicle control embryos. D-F) K02288 treated control embryos. (A, D), *miR26a* morphant (B, E), *miR26a* CRISPRi knockdown (C, F). A’-F’ are threshold adjusted images of *acta2*-EGFP expression. G) Quantification of *acta2* positive cell number on VA and PAAs, within area outlined in A and B. Number of *acta2* positive cells is significantly reduced in K02288 treated embryos as compared to DMSO control. There is no significant decrease in *miR26a* knockdown embryos (two Way ANOVA, N = 3, *miR26a* MO n = 15, Wildtype n = 15, Unpaired t test, ****p< 0.0001 as compared to control, Error Bars = SEM. H) Quantification of length of VA, within area outlined in A and B. Length of VA is significantly reduced in K02288 treated embryos as compared to DMSO control. There is no significant decrease in *miR26a* knockdown embryos (Two Way ANOVA, N = 3, miR26a MO n = 15, Scr.Control n = 15, Unpaired t test, ****p< 0.0001 as compared to control, Error Bars = SEM. VA = ventral aorta, HA = hyoid artery, BA = bulbous arteriosus. (N = 3, 8–9 embryos per treatment group. One Way ANOVA, p< 0.001–0.0001***-****. Scale bar represents 50μm. I-L) pSmad1/59 staining in K02288 treated embryos. Endothelial nuclei (*fli1a*:*nEGFP*; I-L, arrowheads) and p*Smad1*/5/9 (pSmad1, white I’-L’) and overlay (magenta, I”-L”) in 4 dpf Scr. Control and *miR26a* morphants. Solid yellow arrowheads in I”-L” indicate pSmad1 + *fli1a*:*nEGFP* double positive nuclei in the ventral aorta. M) Quantification of total number of *fli1a*:*nEGFP* nuclei in the ventral aorta. N) Quantification of the percent pSmad1; *fli1a*:*nEGFP* double positive nuclei in *miR26a* morphants and *miR26a* CRISPRi embryos. N = 3 experiments, total embryos Scr. Control MO n = 9, miR26a MO n = 9. One Way ANOVA, p< 0.001–0.0001***-****. Scale bar represents 50μm.

Our results showed that *miR26a* knockdown leads to an increased number of *acta2* positive vSMCs on the ventral aorta and upregulation of Smad1 activation in the endothelium. To further investigate the interplay between BMP signalling in endothelial cells and vSMC differentiation, we tested whether the increase in vSMC number and differentiation after loss of *miR26a* could be reversed by blocking endothelial BMP signalling. K02288 is a selective and potent small molecule inhibitor of BMP signalling that blocks Smad1 phosphorylation by type I receptor Activin like kinase 1 (Alk1) and Alk2 [54,55]. We show that *alk1* expression is enriched in endothelial cells at this developmental stage, but not vSMCs (S1C Fig). We selected a time point for drug application when the endothelium of the major blood vessels is patterned [56], but when vSMC coverage of the ventral aorta and PAA is only starting [7]. *Tg(acta2*:*EGFP;kdrl*:*mCherry)* embryos were treated with 15μM K02288 from 52 hpf to 4 dpf. As expected, *miR26a* morphant and *miR26a* CRISPRi treated embryos have significantly more vSMCs than wildtype embryos (Fig 7B, 7B’, 7C, 7C’ and 7G). Wildtype embryos treated with K02288 show a 62% reduction in the total average number *acta2*:*EGFP* positive cells compared to vehicle control alone (Fig 7A’, 7D’ and 7G. 29±1 to 11±3). In *miR26a* knockdown embryos (Fig 7B’ and 7C’), the effects of K02288 were rescued; *miR26a* morphants had a non-significant 17% reduction in vSMC numbers (35±5 to 29±5) and *miR26a* CRISPRi embryos had a non-significant reduction from 39±1 to 34±3 (Fig 7E’, 7F’ and 7G). We also found that BMP inhibition not only affects vSMC number, but also reduces ventral aorta length by 55% in K02288 treated wildtype embryos, from an average 159±2.08 μm to 77±19.5 μm (Fig 7A, 7D and 7H). However, *miR26a* morphants treated with K02288 are rescued and have a ventral aorta length not significantly different than wildtype. *miR26a* CRISPRi embryos showed a smaller rescue and had a 20% decrease in length when treated (Fig 7B, 7C, 7E and 7F, 177±8.6 to 136±5.6). Of note, there was no statistical difference in ventral aorta length between *miR26a* knockdown and control embryos, which supports our finding that endothelial cell number is not affected by loss of *miR26a*.

We next tested whether pSmad1 levels are rescued in K02288 treated *miR26a* knockdown embryos as compared to controls (Fig 7I–7L). Using the endothelial nuclear marker *fli1a*:*nEGFP*, we confirmed that there was no significant difference in endothelial cell number between untreated control and *miR26a* morphants (Fig 7M). In control embryos, treatment with K02288 (Fig 7I, 7I’, 7J and 7J’) significantly reduced the number *fli1a*:*nEGFP* positive cells to 16± 0.2, which is 20% less than controls. Similarly, the proportion of pSmad1 positive/*nEGFP* nuclei also decreased from 43±1% to 28±2% (Fig 7N). Although K02288 treated miR26a morphants have a slight reduction in the total number *fli1a*:*nEGFP* positive cells (Fig 7M), there is no significant decrease in the proportion of pSmad1 positive/*fli1a*:*nEGFP* nuclei (Fig 7K, 7K’, 7L, 7L’ and 7N), and they remain similar to untreated controls. Taken together, our results further suggest that the endothelial cell is a critical site of Smad1-mediated BMP signalling and blocking its activation can significantly affect vSMC coverage. Loss of *miR26a* is able to rescue these defects to maintain both endothelial signalling and vSMC coverage.

## Discussion

**Fig 8:**
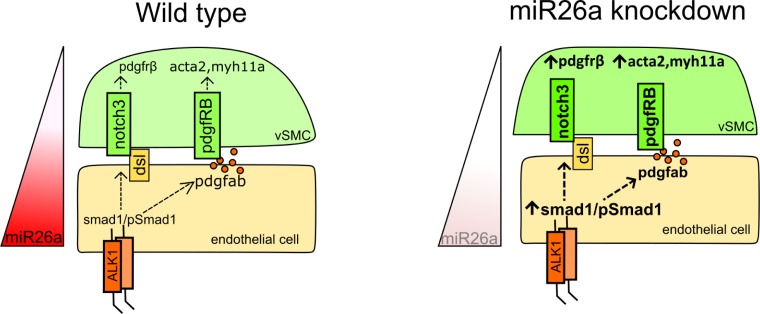
Mechanistic model by which *miR26a* modulates BMP signaling to promote vSMC differentiation via interactions with endothelial cells. *miR26a* modulates vascular stability by directly targeting *smad1*. At developmental stages when smooth muscle appears, the endothelium has active BMP signaling. Loss of *miR26a* results in increased BMP signaling in endothelial cells where Smad1 becomes phosphorylated. Increased pSmad1 in endothelial cells leads to increased differentiation (*acta2* expression) and increased vSMC cell number, while blocking BMP signaling leads to a decrease of both. (Dashed arrows indicated the indirect effect on vSMC marker expression and cell number).

Compromised structural vascular integrity, vessel weakening and rupture (hemorrhage) can result from aberrant BMP signalling [57–59]. Hemorrhage ultimately results from weak endothelial junctions, however defects in mural cell coverage (attachment and ECM secretion) are implicated in the pathological progression of vascular diseases. We show that the endothelium of the ventral aorta in zebrafish has activated pSmad1 at 4 dpf, but that pSmad1 is not detectable in mural cells. At a stage when mature vSMC are normally present, embryos with loss of *miR26a* have upregulation of pSmad1, increased vSMC coverage and a change in vSMC morphology, with no observable changes in the number or morphology of the pSmad1-expressing endothelial cells. We show that inhibition of BMP signalling reduces both vSMC coverage and the length of the ventral aorta while dual *miR26a* knockdown and BMP receptor inhibition leads to a rescue such that animals maintain normal vSMC number, length of the ventral aorta, and vSMC coverage. We therefore suggest that *miR26a* modulates BMP signalling in endothelial cells to control vSMC differentiation via a paracrine mechanism potentially involving Notch and/or Pdgfrβ signalling. We propose that *miR26a* therefore functions *in vivo* to fine tune endothelial signals to the vSMCs (Fig 8).

Studies in cultured vSMC have suggested that *miR26a* controls Smad1-mediated BMP signalling within vSMCs to modulate their phenotype [28]. However, these studies do not address whether the levels of pathway activation *in vitro* are relevant to tissues *in vivo*. Additionally, data collected from *in vitro* culture systems do not address the role of cell to cell communication (autonomous and non-autonomous signalling) that is critical *in vivo* [1,9]. We therefore sought to use an *in vivo* model of vascular development with intact tissue and cellular contexts to assess how loss of *miR26a* and subsequent increases can affect vSMC coverage. We show multiple lines of evidence that suggest that endothelial pSmad1 levels correlate with increased vSMC coverage of blood vessels. Our use of BMP-reporter transgenic fish reveals that during normal development, and under physiological conditions, vSMCs directly contact BRE and pSmad1 positive endothelial cells but have undetectable BRE or pSmad1 signal themselves.

In parallel to loss of *miR26a* resulting in a subsequent increase in *smad1* and vSMC coverage, we also demonstrate that endothelial specific overexpression of *smad1* (smad1^ECOE^) results in increased vSMC coverage. Our data therefore inversely complement the murine knockout models of HHT that have noted reduced vSMC coverage when endothelial Smad1 signalling is reduced. Of note, endothelial specific knockdown of Alk1 or Smad4 leads to a reduction of αSMA/Acta2 coverage on larger arterial vessels. Interestingly, there is a context-dependant shift in vSMC coverage in these studies, as ectopic expression of vSMCs is seen on venous and capillary vessel beds [60]. This hypervascularization was presumed to be in response to increased flow from AVM affected vessels into finer retinal vessels. Similar shifts are seen when BMP9/10 blocking antibodies are used [61]. Our study did not address changes in vSMC coverage in venous beds, but it would be interesting to see if overexpression of *smad1* leads to increased vSMC coverage across both arterial and venous vessel beds.

Our data suggest that the normal function of *miR26a* is to reduce Smad1 protein activation within the endothelium, and indirectly inhibit vSMC differentiation in early development. Treatment with K02288, a potent ALK1/2 inhibitor, significantly reduced both *acta2*-positive vSMC coverage and reduced the length of the ventral aorta. These effects could be rescued by loss of *miR26a*. Thus, we suggest that enhanced Smad1 activation in these embryos compensates for receptor inhibition. ALK1, ALK2 and ALK3 are expressed in both endothelial and vSMCs [17–21], however in zebrafish *alk1* is highly expressed only in the endothelium at 36 hpf [47]. *Violet beauregarde (vbg*^*ft09e*^*) alk1* loss of function zebrafish mutants develop striking cranial vessel abnormalities by 48 hpf due to increased endothelial cell proliferation [19]. *vbg*^*ft09e*^ are also unable to limit the diameter of arteries carrying increasing flow from the heart [27]. Based on our data involving indirect control by endothelial signalling, we would predict there is an additional defect in vSMC recruitment in *alk1* mutants, although this remains to be tested.

Endothelial and mural cells signal through several paracrine pathways to stabilize vessels [62,63]. BMP signalling in endothelial cells activates an axis of BMP/ Notch3/ Pdgf signalling to promote the expression of contractile vSMCs genes such as Acta2 and Myh11a in *in vitro* co-culture systems [64]. Specifically, BMP9 signalling via endothelial cells induces NOTCH3 in vSMCs, which in turn induces expression of Pdgfrβ and maintains the proper response to Pdgf ligands [17,65]. There is evidence that mouse MiR26a is modulated by Pdgf-BB signalling [45]. For instance, neointimal hyperplasia results in elevated levels of Pdgfbb associated with upregulation of MiR26a and accumulation and proliferation of vSMC at sites of injury. Furthermore, treatment of primary mouse aortic vSMCs with MiR26a mimic drives cells to a synthetic vSMC state [45]. We found that *notch3*, *pdgfrβ* and contractile vSMC markers were significantly increased in *miR26a* knockdown embryos, suggesting that increases in endothelial Smad1 in zebrafish may be transmitted to vSMCs through a BMP/ Notch3/ Pdgf signalling axis. Pdgf ligands are primarily released by endothelial cells, and we observe an increase in *pdgfba* in *miR26a* morphants, providing a potential mechanism by which active BMP signaling in endothelium can recruit and induce vSMC differentiation via paracrine non-autonomous signalling pathways.

While we found increased differentiation of vSMCs at the later stage 4 dpf time point, at 48 hpf loss of *miR26a* results in hemorrhage. The 48 hpf to 4 dpf window is a common window for vascular instability phenotypes to emerge in zebrafish [3,66–68]. BMP signalling is initiated in endothelium at this time point and perturbations can affect endothelial cell junction development [63]. We have previously shown mural cells present around vessels by 48 hpf, although they are mesenchymal and immature [3]. These cells express *pdgfrβ* but have no expression of mature vSMC markers [69], suggesting the 48 hpf time point represents a critical window for vascular mural cell attachment to endothelium and differentiation to a mature phenotype. It is paradoxical then that we see increased maturation of vSMCs at 4 dpf when *mir26a* is reduced. We suggest that the altered receptor and ligand expression in *miR26a* morphants may promote morphological change towards maturation, but may not regulate all aspects of maturation, leading to destabilization. For instance aberrant ECM deposition would not be visible in our assays and could lead to vascular instability at the earlier time points [63].

As critical modulators of vascular cell function and with roles in cell differentiation, contraction, migration, proliferation and apoptosis, miRs are attractive targets of therapeutic treatments aimed at modulating the vSMC phenotypic switch. Specific to TGF-β/BMP signalling, the *miR-145/143* family has direct involvement in SMC differentiation by repressing the Klf4 to induce a contractile morphology and reduced rates of proliferation [40]. *miR-21* controls vSMC differentiation through cross-talk with *miR-143/-145* [35] and by mediating TGF-β/BMP induction to promote *miR-21* cleavage to its mature form and a more contractile phenotype (Fig 5). *miR26a* is unique in this group in that it represses smooth muscle differentiation, likely via a paracrine signalling from endothelial cells. As drug delivery to the endothelium is relatively straightforward, modulation of *miR26a* might be therapeutically useful for post-transcriptional control of key genes involved in vSMC phenotypic switching.

## Materials and methods

### Ethics statement

All animal procedures were approved by the University of Calgary Animal Care Committee (AC17-0189). Anesthesia and euthanasia used MS-222 (Tricaine) at 10–40 mg/L.

### Zebrafish maintenance and husbandry

Zebrafish (*Danio rerio)* embryos were collected and incubated at 28.5°C in E3 embryo medium and staged in hours post-fertilization (hpf) or days post fertilization (dpf). Endogenous pigmentation was inhibited from 24 hpf by the addition of 0.003% 1-phenyl-2-thiourea (PTU, Sigma-Aldrich, St. Louis, MO) in E3 embryo medium. The fluorescent transgenic endothelial mCherry-expressing *Tg(kdrl*:*mCherry)*^*ci5*^, GFP-expressing *Tg(kdrl*:*EGFP)*^*la116*^ report endothelial expression and *Tg(fli1a*:*nEGFP)*^*y7*^ [19] reports EGFP cDNA fused to a nuclear localization sequence in endothelial nuclei. *Tg(acta2*:*GFP)*^*ca7*^ and *Tg(acta2*:*mCherry)*^*ca8*^ report smooth muscle expression [7]. BMP-reporter fish *Tg(BRE-AAVmlp*:*EGFP)*^*mw29*^ [BRE:EGFP] report active BMP signaling [46].

### Morpholino knockdown, CRISPRi and mRNA overexpression

Both MO and mimic were injected into one- to four-cell stage embryos within recommended dosage guidelines [50,70]. Injected doses were 1ng/ embryo for *miR26a* MO, Scrambled (Scr.) control, *miR26a*, and *smad1* MO. Morpholinos (MO) were obtained from Gene Tools LLC (Corvallis, OR, USA). *mir-26a* MO blocks the mature microRNA (5 AGCCTATCC*TG*GATTACT*TG*AAC-3’), *miR26a* Scrambled control has 6bp mismatch (5’-ACCGTATCG*TG*CATTACTTCAAC-3’), and *smad1* MO blocks Smad1 translation (5’-AGGAAAAGAG*TG*AGG*TG*ACATTCAT-3’) [53]. For rescue experiments, embryos were first injected with *miR26a* MO and then *smad1* MO. To control for non-specific neural cell death that occurs from nonspecific activation of p53 with morpholinos, a standard p53 MO was co-injected with high dose morpholino to establish dosage curve. Hsa *miR26a* miRIDIAN mimic was obtained from Dharmacon (Chicago, IL) and injected in a dose of 3ng/ embryo.

For CRISPRi mediated knockdown of *miR26a*, sgRNA were designed using CHOPCHOP [71,72] to target the seed sequence of *miR26a* family members, to reduce *miR26a* processing. *MiR26a-1*, *miR26a-2*, *miR26a-3*, are independent genes located on different chromosomes. *miR26b* differs by one nucleotide. To generate sgRNA we followed a method established by [73]. 10 μmol of forward primer (5’ TAATACGACTCACTATAGGATCCT GGATTACTTGAACCAGTTTTAGAGCTAGAA-3′) and 50 μmol of a universal reverse primer (5′AAAAGCACCGACTCGGTGCCACTTTTTCAAGTTGATAACGGACTAGCCTTATTTTAACTTGCTATTTCTAGCTCTAAAAC-3′), (IDT Oligos, Coralville, Iowa were annealed and filled in [72], purified (Qiagen PCR purification kit) and *in vitro* transcribed (T7 mMESSAGE mMACHINE kit, Ambion, Austin, TX. Zebrafish codon optimized dCas9 plasmid [74] was linearized with XbaI and *in vitro* transcribed using Ambion Maxi Kit (Life Technologies Inc., Burlington, ON), and RNA purified using an RNeasy Mini Kit (Qiagen, Hilden, Germany). Zebrafish embryos at the one-cell stage were injected with 200pg of a solution containing 75 ng/ μl of sgRNA with 150 ng/ μl of Cas9 mRNA. For overexpression of *smad1*, mRNA was *in vitro* transcribed as described (McReynolds et al. 2007; gift from Todd Evans Lab) using mMessage mMachine (Life Technologies Inc., Burlington, ON). 40 pg of mRNA was injected per embryo at the 1 cell stage.

### Plasmid construction

For endothelial specific overexpression, *smad1* was amplified from zebrafish cDNA using primers that incorporate attb1/b2 recombination sites (5’-GGGGACAAGTTTGTACAAAAAAGCAGGCTTCACCATGAATGTCACCTCACTCTTTTCC-3’ and 3’- GGGGACCACTTTGTACAAGAAAGCTGGGTGCTAGGACACTGAAGAAATGGGGT-5’ and inserted into pDONR221 to create a pME-*smad1* vector. Three way Tol2 gateway cloning [75] was used to insert *smad1* downstream of the *kdrla* promoter to achieve a TolCG2:*kdrla*:*smad1* vector. One-cell stage zebrafish embryos were injected with a solution consisting of 5–20 ng/ μl *kdrla-smad1* plasmid and 50 ng/ μl transposase mRNA.

For the *in vivo* sensor test, *smad1* 3′UTR forward and reverse oligos (IDT) were designed incorporating BamHI and Bsrg1 sites using the prediction software TargetScan [76] for miR26 targets within the 3’UTR of zebrafish *smad1 (*underlined). (5’GCGTGTACACCGGATGACTAGAGGGTTAGGTTGTGTACTACTTGAAGGCAGTTTGTTAGGGTGGGGGTCATCGAATCTGGCTGAAGAGTCCTCAGTTTTCAGCCCGTGAGAATCTGGAAGATACTTGACAACTCTGTGGCCGGATCCATA-3’ and 3’- TATGGATCCGGCCACAGAGTTGTCAAGTATCTTCCAGATTCTCACGGGCTGAAAACTGAGGACTCTTCAGCCAGATTCGATGACCCCCACCCTAACAAACTGCCTTCAAGTAGTACACAACCTAACCCTCTAGTCATCCGGTGTACACGC-5’). Oligos were digested and ligated into the p3E-polyA vector. This construct was then recombined into pDestTol2pA2 by Gateway cloning to achieve a CMV-SP6 promoter upstream of EGFP: *smad1* 3′UTR:EGFP or a control EGFP: p3E-polyA 3′UTR. Sensor mRNA and mCherry mRNA were in vitro transcribed from the pCS2 Gateway compatible vector (39) by using the mMessage Machine SP6 kit (Ambion). One-cell zebrafish embryos were injected with 150 pg sensor mRNA and 100 pg mCherry mRNA. When applicable, *miR26a* MO or miRNA mimic were added. Live embryos were imaged with an identical exposure time at 24 hpf (n = 10/group). The average pixel intensity for fluorescence was measured as described (17)

### Cell sorting, RNA isolation and RT-qPCR

For FACS analysis ~200 embryos were collected from 4 dpf Tg(acta2:EGFP;kdrl:mCherry) fish. Embryos were anesthetized with 0.4% Tricaine (Sigma) and heads dissected and pooled. Single cell dissociation was performed according to Rougeot et al. 2014. Briefly dissected embryo heads were washed once with calcium-free Ringers Solution and gently triturated 5–10 times before dissociation solution was added and incubated in a 28.5°C water bath with shaking and periodic trituration for 45 min. The reaction was stopped, centrifuged and resuspended in Dulbecco’s Phosphate-Buffered Saline (GE Healthcare Life Sciences, Logan, Utah, USA, centrifuged and resuspended in fresh resuspension solution. The single cell suspension was filtered with 75 μm, followed by 35 μm filters. Cells were then sorted with a BD FACSAria III (BD Bioscience, San Jose, USA) and collected.

Total RNA from 48 hpf whole embryos, 4 dpf dissected embryo heads or FACS sorted cells was isolated using the miRNeasy Mini Kit (Qiagen). For microRNA RT-qPCR, 5 ng of total RNA from each sample was reverse transcribed using the miRCURY LNA Universal RT cDNA Synthesis Kit and expression assayed using the miRCURY LNA Universal RT microRNA PCR System (Qiagen). Primers were ordered for miR26a-5p (MIMAT0000082, Target sequence: UUCAAGUAAUCCAGGAUAGGCU), and, expression levels normalized to that of miR-103a-3p (MIMAT0000425, Target sequence: CAGUGCAAUGUUAAAAGGGCAU) or miR122 (MIMAT0000421, Target sequence: AGCUACAUUGUCUGCUGGGUUUC for miRNA expression)

For gene expression, zebrafish specific Taqman assays (Thermo Fisher Scientific, Waltham, Massachusetts, USA) were used: smad1 (Cat# 4351372, Clone ID: Dr03144278_m1), acta2 (4331182, Dr03088509_mH), myh11a (444889, Dr03141711_m1), pdgfrβ (4441114, ARKA4GC), pdgfba (4441114, ARWCXGT), nothch3 (4448892, Dr03432970_m1) and normalized to β-actin (4448489, Dr03432610_m1). 500 ng of total RNA from each sample were reverse transcribed into cDNA using and assayed using according to manufacturer’s protocols in a 5ng/ 10ul final reaction using TaqMan Fast Advanced Master Mix (Thermo Fisher). Reactions were assayed using a QuantStudio6 Real-time system (Thermo Fisher).

The ΔΔCt method was used to calculate the normalized relative expression level of a target gene from triplicate measurements. Experiments were repeated independently at least three times, unless stated otherwise.

### Small molecule inhibition

K02288 was used at a dose of 15μM (SML1307, Sigma). DSMO (D8418, Sigma) was used as a vehicle and control. Drug stocks were heated for 20 min at 65C and then diluted in E3 embryo medium. Drug or control was applied to the media from 52 hpf until 4 dpf. Embryos were grown at 28.5C in the dark until imaging, and drug changed once.

### In situ hybridization and immunostaining

All embryos were fixed in 4% paraformaldehyde in PBS with 0.1% Tween-20 at 4°C overnight, followed by 100% methanol at −20°C. Digoxigenin (DIG)-labeled antisense RNA probes were used for in situ hybridization. Probes for *smad1* (construct described by [53]) *sm22a*, *acta2*, *myh11a* were synthesized from PCR fragments previously described [7,48]. Probes were synthesized by using SP6 or T7 RNA polymerase (Roche, Basel, Switzerland). *miR26a* double-DIG-labeled LNA probe was obtained from Exiqon, (Copenhagen, Denmark. In situ hybridization was performed as described [32]using a Biolane HTI robot (Holle and Huttner AG, Tubingen, Germany). For microRNA in situ hybridization, a double-DIG-labeled Locked Nucleic Acid (LNA) probe (Exiqon) was used to detect the mature miR26a in whole-mount embryos as recommended by the manufacturer with the modification that hybridization was at 54°C.

For wholemount immunostaining an antigen retrieval protocol optimized from [77] was used. Briefly embryos are hydrated into PBST, washed twice with 15 mM Tris–HCl pH 9.5, 150 mM EDTA and then heated in 15 M Tris–HCl pH 9.5, 150 mM EDTA at 70°C for 15 min. Embryos are then washed 3 times in PBST at room temperature and incubated in 10% normal sheep serum in PBST with 1% triton block and incubated for at least 48 hours at 4°C in primary antibody. Phospho-SMAD1/5/9 (pSMAD1/5/9) was detected with Rabbit anti-Phospho-Smad1 (Ser463/465)/Smad5(Ser463/465)/Smad9(Ser426/428) (1:400; Cell Signaling Technology, Danvers, Massachusetts, USA), GFP was detected with mouse anti-GFP antibody, JL8 (1:500, Clontech, Mountain View, California, USA) and detected with Alexafluor 647 or 488 secondary antibodies for 1 hour at room temp in 5% normal sheep serum in PBST with 0.1% triton (1:500; Invitrogen Molecular Probes).

### Imaging and data analysis

For imaging, embryos were immobilized in 0.004% Tricaine (Sigma) and mounted in 0.8% low melt agarose on glass bottom dishes (MatTek, Ashland, MA). Confocal images were collected on a Zeiss LSM 700 inverted microscope. Image stacks were processed in Zen Blue and are presented as maximal intensity projections and analyzed using FIJI/ImageJ [78] For cell counts images were converted to 16-bit using ImageJ and the threshold adjusted to allow counting of cells over a region of the VA from the anterior bulbous arteriosus to the most anterior PAA.

To measure intensity, total cell fluorescence (CTCF) was calculated using the formula: CTCF = Integrated Density—(Area of selected cell X Mean fluorescence of background readings). The area for measurement was gated by tracing the aorta from bulbous where the bulbous arteriosus merges with the ventral aorta to the distal tip of the ventral aorta or to the bifurcation point of the ventral aorta using the free form drawing tool, whichever was shorter [56].

For measurement of vSMC cell heights, measurements were made from the endothelial *kdrla*:EGFP expression to the highest point of the vSMC. 8 measurements were taken for each sample where possible. Ventral head measurements were taken from the ventral aorta and the aortic arch arteries. Measurements represent mean vessel diameter ± standard deviation in micrometers.

### Statistical analysis

Distribution of data points are expressed as mean ± standard error of the mean (S.E.M.), or as relative proportion of 100% as mentioned in the appropriate legends. Depending on the number of the groups and independent factors, student's t-tests, one-way or two-way analyses of variance (ANOVA) with non-parametric tests were used as indicated in the figures. Two treatment groups were compared using Student’s t-test, using Welch’s correction. Three or more treatment groups were compared by one- or two-way ANOVA followed by post hoc analysis adjusted with a least significant-difference correction for multiple comparisons using GraphPad Prism version 7.00 (La Jolla California USA). Results were classed as significant as follows: *P < 0.05, **P < 0.01, and ***P < 0.001.

## Supporting information

S1 Fig p*Smad1* is observed in endothelial cells. A) Schematic of FACS sorting strategy for acta2:EGFP+ and kdrl:mCherry+ cells from 4 dpf *Tg(acta2*:*EGFP;kdrl*:*mCherry)* dissected embryo heads. B) Expression level of *miR26a* in acta2:EGFP+ and kdrl:mCherry+ isolated by FACS (n = 2). C) Expression level of *smad1*, *acvrl (alk1)* and *acta2* in acta2:EGFP+ and kdrl:mCherry+ isolated by FACS (n = 2). RT-qPCR analysis of values represent mean ± SEM, n = biological replicates. D) Wholemount immunohistochemistry of pSmad1/5/9 in *Tg(acta2*:*EGFP)*^*ca7*^*; Tg(kdrl*:*mCherry)*^*ci5*^ shows nuclear staining of pSmad1 in endothelial cells. pSmad 1/5/9 is observed in endothelium (red) but not smooth muscle (green) of the hyoid artery and afferent branchial arches. Magenta asterisk indicates pSmad1/5/9 stain; arrows indicate smooth muscle cells. Scale bar represents 50μm.
(EPS)

S2 Fig *miR26a* overexpression decreases *smad1* expression. A) *EGFP*:*smad1* sensor assay showing *EGFP-smad1* sensor expression vs mCherry control. B) Quantification of EGFP fluorescence in sensor assay as compared to controls. Student's two-tailed t-test *p< 0.05, N = 3, total of 9 embryos per group, Error Bars = SEM, Scale bar represents 50μm. C) RT-qPCR of relative expression of *miR26a* in *miR26a*-mimic injected embryos at 48 hpf. Values are means of 3 replicates and normalized to *miR122*; Unpaired t test, *p< 0.01 as compared to control; Error Bars = SD. D) *miR26a* mimic injected embryo with mild dorsalization phenotype, heart edema (arrowhead), dorsal axis defects (arrows) and poor circulation (arrowhead at tail) at 48 hpf. E) Whole mount in situ hybridization staining for *smad1* at 48 hpf in uninjected control, negative control mimic and *miR26a* injected embryos. There is decreased expression of *smad1* in *miR26a* mimic injected embryos (boxes and arrow).
(EPS)

S3 Fig miR26a knockdown embryos have increased endothelial p*Smad1*. Ventral view confocal projections of 4 dpf Tg(*kdrl*:*mCherry; fli1a*:*nEGFP* ventral aorta (dotted outline. A-D) Endothelial cytoplasm (kdrl:mCherry) and endothelial nuclear (fli1a:nEGFP) staining. E-H) fli1a:nEGP endothelial nuclear stain. I-L) pSmad1/5/9 staining. M-P) overlay image, in 4 dpf Scr. Control (A,E,I,M), *miR26a* MO (B,F,J,N), dCas9 control (C,G,K,O) and *miR26a* CRISPRi (D,H,L,P). Q) Average number of fli1a:nEGFP nuclei. R) Average percentage pSmad1/fli1a:nEGFP double positive nuclei. Scale bar represents 50μm.
(EPS)

S4 Fig *miR26a* morpholino results in dose-dependent increase in hemorrhage and *smad1*. A) RT-qPCR of relative expression of *miR26a* at 48 hpf in *miR26a* MO (28 ng/ embryo). Values are means of 3 replicates and normalized to *miR122*, n = 3: UIC vs. Neg. ctl. MO (Scr.Control MO) p = 0.16. UIC vs. Pre-*miR26a* MO: p < .0001. UIC vs. *miR-26a* mature MO: p = 0.0002. RT-qPCR analysis of values represent mean ± SEM, n = 2 biological replicates. B) Whole-mount in situ hybridization staining for *smad1* at 48 hpf shows increased expression of *smad1* at higher dose of *miR26a* MO. C) Representative images of wildtype (uninjected) and high dose *miR26a* morpholino-injected embryos showing normal body axis, but with hemorrhage and mild hydrocephalus. D) Average rates of hemorrhage for *miR26a* MO at 28 ng/ embryo and 6 ng/ embryo (Student's two-tailed t-test, ****p< 0.0001 as compared to WT, Error Bars = SEM. N = 5, n total *miR26a* MO 28 ng = 488, 6 ng = 501 and Wildtype = 535).
(EPS)

S5 Fig Changes in vascular smooth muscle marker gene expression in *miR26a* morphant and mimic embryos. A-D) Whole-mount in situ hybridization staining for *acta2* and *myh11a* at 4 dpf shows increased expression in *miR26a* knockdown embryos in the aortic arches and pharyngeal region. E-J) Whole-mount in situ hybridization staining for *acta2* and *sm22α* at 4 dpf shows decreased expression in *miR26a* mimic injected embryos.
(EPS)

S6 Fig Smad1 overexpression. A) Phenotypes observed at 48 hpf with increasing doses of *smad1*^*ECOE*^. B) Quantification of phenotypes, Student's two-tailed t-test **-****p< 0.005–0.00005 as compared to control. Error Bars = SEM. N = 3, n total smad1^-ECOE^ 5 ng, n = 60, 10 ng, n = 61, 20 ng, n = 63 and control n = 72. C) Ventral view confocal projections of the 4 dpf ventral aorta of Tg(BRE:EGFP) embryos. D) Average number of acta2:mCherry cells, BRE intensity and vSMC coverage.
(EPS)

S1 File Numerical supporting information. File of raw data underlying graphs.
(XLSX)
